# Infectivity of amphibian chytrid fungi requires metalloprotease-driven spore development and germ tube formation

**DOI:** 10.1186/s12866-026-04896-x

**Published:** 2026-03-25

**Authors:** Elin Verbrugghe, Frank Pasmans, An Martel

**Affiliations:** Wildlife Health Ghent, Department of Pathobiology, Pharmacology and Zoological Medicine, Faculty of Veterinary Medicine, Ghent University, Merelbeke, B-9820 Belgium

**Keywords:** Chytridiomycosis, *Batrachochytrium dendrobatidis* (Bd), *Batrachochytrium salamandrivorans* (Bsal), Amphibian pathogens, Metalloproteases, Protease inhibitors, Spore development, Germ tube formation, Host-pathogen interactions, Fungal pathogenicity

## Abstract

**Background:**

Chytridiomycosis, caused by the fungi *Batrachochytrium dendrobatidis* (Bd) and *B. salamandrivorans* (Bsal), drives global amphibian declines. Infection of a susceptible host begins when zoospores attach to the skin and extend germ tubes to penetrate the epithelial tissue. Proteases, particularly metalloproteases, are thought to degrade extracellular matrix components, facilitating tissue invasion, but their roles in spore development and early host colonization remain unclear.

**Results:**

We demonstrated that metalloproteases are rapidly secreted by zoospores and are critical for the transition from attachment to germ tube outgrowth and subsequent spore development, important steps in infectivity. Two protease inhibitor mixtures, PI 8340 and PI 8215, were tested in vitro, with PI 8215 containing the metalloprotease-specific inhibitor 1,10-Phenanthroline. PI 8340 had minimal effects on zoospore viability at concentrations up to 0.33% (v/v) for Bd and 1% (v/v) for Bsal. In contrast, PI 8215 impaired spore viability in a dose-dependent manner, with significant effects from 0.0025% (v/v, 12.5 µM 1,10-Phenanthroline) in Bd and 0.005% (v/v, 25 µM 1,10-Phenanthroline) in Bsal. Sublethal concentrations of PI 8215 (0.005% v/v) and 1,10-Phenanthroline (25–50 µM) suppressed germ tube formation, which resumed once the inhibitors were removed. Protease activity assays confirmed that metalloproteases are secreted during early spore stages, with activity detectable as early as 2 h after spore collection, highlighting their role in initial spore development. Metalloprotease activity depends on specific metal cofactors, as growth inhibition by 1,10-Phenanthroline could be partially rescued in Bsal with Zn²⁺ (100 µM) or Mg²⁺ (20 mM). In Bd, recovery was limited and largely independent of cation supplementation. *Ex vivo* infection of *Pleurodeles waltl* skin confirmed the in vitro findings, as a lethal concentration (100 µM) significantly reduced Bsal loads after five days, whereas a sublethal concentration (20 µM) had no detectable effect, possibly due to protective cations in host tissue.

**Conclusions:**

Metalloproteases are critical for spore development. Inhibition of these enzymes stalls germ tube formation and spore development, highlighting their central role in the earliest stages of chytrid development and suggesting their importance for infectivity.

**Supplementary Information:**

The online version contains supplementary material available at 10.1186/s12866-026-04896-x.

## Background

Chytridiomycosis, caused by *Batrachochytrium dendrobatidis* (Bd) and *B. salamandrivorans* (Bsal), has had a devastating impact on global amphibian populations, contributing to widespread declines and extinctions [34]. These fungal pathogens primarily infect the keratinized epithelial tissues of amphibians, disrupting skin function and causing lethal infections [3, 22]. Both Bd and Bsal employ an active invasion mechanism in which zoospores adhere to the host skin and initiate infection through germ tube protrusion, allowing direct penetration into host tissue [39, 41]. This process is notably independent of conventional host actin dynamics, suggesting a pathogen-driven mode of entry that bypasses host cellular defenses [41]. The active penetration mechanism is supported by the presence of an expanded repertoire of protease-encoding genes in both the Bd and Bsal genomes [14, 18, 19, 42]. Activity-based studies, together with gene expression studies, support the idea that proteases are key during the early stages of zoospore colonization and penetration, facilitating enzymatic digestion of the epithelial surface [8, 26, 32, 33, 36, 14, 42]. However, the exact role of these proteolytic enzymes in Bd and Bsal pathogenesis remains to be elucidated.

Among the proteases identified in Bd and Bsal, metalloproteases are particularly notable due to their extensive genomic expansion [42]. Relying on divalent metal cations such as Zn²⁺ for their activity, these enzymes are central to fungal virulence, and fluctuations in ion availability can affect metalloprotease function and, consequently, infection efficiency. Metalloproteases are classified into two main subfamilies: deuterolysin (M35) and fungalysin (M36) families, two major types of zinc metalloproteinases secreted by pathogenic fungi, with the M36 subfamily being particularly adept at hydrolyzing extracellular matrix proteins [18, 21]. In Bd and Bsal, there is a marked expansion of M36 metalloprotease genes, with Bsal showing the largest expansion of M36 metalloproteases in any species [42]. Like other pathogenic fungi, such as *Candida albicans*, *Aspergillus fumigatus* and *Cryptococcus neoformans*, which rely on Zn²⁺ to fuel virulence-associated metalloproteases and must compete with the host for this essential micronutrient [7, 23, 29], Bd and Bsal are assumed to depend on Zn²⁺ availability on the basis of the extensive metalloprotease gene expansion observed. Gene expression studies have shown that some M36 metalloprotease genes are upregulated during the later stages of infection in heavily infected animals, whereas others are activated early, likely upon initial contact with the host skin [14]. These findings suggest that different metalloproteases may play stage-specific roles.

In this study, we investigated the role of metalloproteases in Bd and Bsal mechanisms of spore maturation and host colonization via a combination of protease activity assays, growth assays, inhibition experiments, and in vitro infection models. Two protease inhibitor (PI) mixtures, PI 8340 and PI 8215, were employed to target different classes of proteases. PI 8340 includes inhibitors for serine proteases (AEBSF, 104 mM), aminopeptidases (Bestatin, 4 mM), cysteine proteases (E-64, 1.4 mM), serine and cysteine proteases (Leupeptin, 2 mM), and acid proteases (Pepstatin A, 1.5 mM). PI 8215 has a similar composition but uniquely incorporates 1,10-Phenanthroline (500 mM), a metalloprotease inhibitor, which distinguishes it from PI 8340. To specifically evaluate the impact of metalloprotease inhibition, 1,10-Phenanthroline was also tested separately. Furthermore, we assessed the protective effects of divalent cations (Zn²⁺, Ca²⁺ and Mg²⁺) in counteracting metalloprotease inhibition and explored species-specific differences between Bd and Bsal. Finally, the effect of 1,10-Phenanthroline on Bsal colonization was evaluated using an *ex vivo Pleurodeles* (*P*.) *waltl* skin infection model. Dissecting the role of metalloproteases in Bd and Bsal spore initiation provides insight into the critical early steps of amphibian infection.

## Methods

### Bd and Bsal growth conditions

Bd strain JEL 423 (kindly provided by Dr. Joyce Longcore University of Maine, USA) [13] and Bsal type strain (AMFP 13/01) [22] were cultured in tryptone-gelatin hydrolysate-lactose (TGhL) broth and incubated in 75 cm^2^ cell culture flasks at 20 °C for Bd and 15 °C for Bsal for 5–7 days. We collected zoospores from a fully grown culture containing mature sporangia. Upon release of the zoospores, the culture medium was collected and filtered through a sterile mesh filter with a pore size of 10 μm (PluriSelect, Leipzig, Germany). The resulting flow-through, enriched with zoospores at > 90% purity, was utilized as the zoospore fraction for further experimentation. Zoospore viability and mobility were confirmed via light microscopy.

### Reagents and chemicals

The role of proteolytic enzymes was examined via PI cocktails (Sigma Aldrich: P8340 and P8215) and 1,10-Phenanthroline as a metalloprotease inhibitor. Prior to use, the inhibitors were thawed and diluted in the appropriate culture medium.

### Cell culture media

Three distinct media compositions were employed in this study. For the routine growth and maintenance of A6 cells, complete growth medium comprising 74% NCTC 109 medium, 15% distilled water, 10% fetal bovine serum (FBS), and 1% of a 10,000 U/ml penicillin-streptomycin solution was used. Upon exposure of A6 cells to Bd or Bsal, L-15-based cell medium was used. To facilitate the mobility of the zoospores during the initial contact phase, 40% infection medium for Bd and 30% infection medium for Bsal were utilized, comprising 40% or 30% L-15 medium, 50% or 60% distilled water, and 10% FBS. Following the initial 2-hour period, during which the zoospores actively migrated toward the A6 cells, the medium was replaced with 70% infection medium consisting of 70% L-15 medium, 20% distilled water, and 10% FBS.

### Cell culture

The Xenopus laevis kidney epithelial cell line A6 (ATCC-CCL 102) was cultured in 75 cm2 cell culture flasks and maintained in complete growth medium. The cells were incubated at 26 °C with 5% CO2 until they reached confluence [30]. Upon confluence, the cells were detached via trypsin, washed with 70% Hanks’ balanced salt solution without Ca2+ and Mg2+ (HBSS-) by centrifugation for 5 min at 1500 rpm, and then resuspended in the appropriate cell culture medium for invasion assays.

### Toxicity of PI on Bd and Bsal zoospores and A6 cells

To evaluate the impact of PI 8340, PI 8215 and 1,10-Phenanthroline on chytrid viability, Bd (1-2.5 × 10⁵) and Bsal (1-7.5 × 10⁵) zoospores were seeded in TGhL medium containing varying concentrations of PIs in 24-well plates. As controls, zoospores were treated with TGhL alone or TGhL supplemented with the highest DMSO concentration present in the PI treatments to account for solvent effects. In addition, heat-killed zoospores were included as a positive control. Zoospores were heat-killed by incubation at 100 °C for 10 min prior to seeding. Following 24 h of exposure, the medium was replaced with 1 ml of control TGhL medium, and the cultures were further incubated for 4 days. On day 5 post zoospore inoculation (p.i.), Bd and Bsal were harvested from the bottom of the well via a 1 ml pipette tip, combined with the supernatant, and centrifuged for 5 min at 3000 rpm. The resulting pellet was resuspended in 50 µl of PrepMan Ultra reagent, heated for 10 min at 100 °C, and allowed to cool to room temperature for 2 min. Subsequently, the tubes were centrifuged for 2 min at 13 000 rpm, and total Bd and Bsal counts in the supernatants (genomic equivalents (GEs) per well) were determined via quantitative PCR [4, 5]. All qPCR reactions were performed in duplicate using 1/10 diluted DNA. GEs were determined based on a standard curve ranging from 1 000 to 0.1 GE of zoospores per real-time PCR mixture [4, 5], and the number of GEs per well was calculated by correcting for the 1/10 dilution and the total DNA volume. All treatments were tested in at least three independent experiments (biological replicates) with at least three technical replicates. In the figures, data points represent the means of the technical replicates for each biological replicate.

To evaluate the role of proteases in Bd and Bsal infection, the toxicity of the PIs was also assessed on A6 cells. Therefore, we conducted a neutral red assay according to previously established protocols [31]. Briefly, A6 cells were seeded in 96-well microplates at a density of approximately 2 × 10^5^ cells per well in growth medium and allowed to attach overnight at 26 °C with 5% CO2. The cells were subsequently exposed to varying concentrations of PI in 70% infection medium for 24 h. Negative controls comprised cells treated with 70% infection medium, whereas the positive controls were treated with 1% Triton. Cytotoxicity was assessed by adding 200 µL of freshly prepared neutral red solution (33 µg/mL in 70% L-15 medium without phenol red) prewarmed to 26 °C to each well, followed by incubation at 26 °C for an additional 2 h. After the cells were washed twice with 70% Hanks’ balanced salt solution with Ca^2+^ and Mg^2+^ (HBSS+), 200 µL of extraction solution (ethanol/Milli-Q water/acetic acid, 50/49/1, v/v/v) was added to each well, and the plate was shaken for 10 min. Absorbance was measured at 540 nm via a microplate spectrophotometer (Multiscan Go, Thermo Scientific). Relative viability, expressed as a percentage of the negative control (considered 100%), was calculated via the following formula: 100 × ((a − b)/(c − b), where a = OD_540_ from wells treated with CD, b = OD_540_ from blank wells, and c = OD_540_ from untreated control wells. All treatments were tested in at least three independent experiments (biological replicates) with at least three technical replicates. The EC_50_ of PI for A6 cells calculated by fitting a dose-response curve using GraphPad Prism software 8.4.3. In the figures, data points represent the means of the technical replicates for each biological replicate.

### Influence of PI on Bd and Bsal early infection dynamics in A6 cells

The influence of PI 8340 (0.2% v/v), PI 8215 (0.005% v/v), or 1,10-phenanthroline (25 µM) on the early infection steps of Bd and Bsal in A6 was visualized using the in vitro infection models described in Verbrugghe et al. [40] and Verbrugghe et al. [41]. In short, 10^5^ A6 cells were seeded per well in 24-well culture plates containingcollagen-coated glass coverslips and allowed to attach overnight at 26 °C with 5% CO2. Following attachment, the cells were washed three times with 70% HBSS + and subsequently inoculated with Bd or Bsal zoospores at a multiplicity of infection (MOI) of 1:10 in infection medium (40%-30%) with or without protease inhibitors. The cells were then incubated for 2 h at 20 °C (Bd) or 15 °C (Bsal). After incubation, non-attached zoospores were removed by gently washing the cells three times with 70% HBSS+. The cells were then supplemented with infection medium (70%) with or without the same protease inhibitors for an additional 22 h (total exposure time: 24 h p.i.). After 24 h p.i., the medium was replaced with inhibitor-free infection medium (70%) for all conditions. On day 3 p.i., fresh inhibitor-free infection medium (70%) was added. Chytrid-A6 cell interactions were visually assessed using two complementary staining protocols. In the first protocol, infected cells were stained with 9 µM CellTrackerTM Green CMFDA for 45 min at 15 °C with 5% CO2, followed by gentle washing with 70% HBSS+. Extracellular chytrid was visualized by incubating the infected cells with Calcofluor White stain (50 µg/ml in 70% HBSS+) for 10 min, followed by two washes with HBSS+. Cells were then fixed with 3.7% paraformaldehyde for 10 min, permeabilized with 0.1% Triton X-100 for 2 min and incubated for 60 min with a polyclonal antibody against Bd (JEL423) or Bsal (type strain AMFP13/1) produced in rabbit (1/1000) [37]. After washing, samples were incubated with a monoclonal goat anti-rabbit Alexa Fluor 568 antibody (1/750 dilution, Life Technologies) to visualize chytrid, then washed, mounted with ProLong™ Gold antifade mountant, and analyzed via fluorescence microscopy. In the second protocol, infected cells were stained with Phalloidin Texas Red^®^-X (1:100 in 70% HBSS+) for 1 h to visualize the actin cytoskeleton. Extracellular chytrid was stained with Calcofluor White as described above, and the cells were fixed, permeabilized and incubated with the same polyclonal chytrid antibody. However, here the secondary antibody was a monoclonal goat anti-rabbit Alexa Fluor 488 (1/250 dilution, Life Technologies) to allow combination with the Texas Red cytoskeleton stain. Samples were then washed, mounted, and analyzed similarly. Sham-infected cells served as negative controls. The presence of Bd and Bsal invasion and maturation in A6 cells was assessed qualitatively, based on whether infection was visibly detectable, without quantitative measurement.

### Influence of PI on Bd and Bsal germ tube development

Based on toxicity experiments, PI 8215 and 1,10-Phenanthroline at sublethal concentrations seem to suppress the development and possibly the germination of Bd and Bsal. We therefore examined whether the germ tube formation of zoospores treated with PI 8340, PI 8215 or 1,10-Phenanthroline was affected. To link the results to the in vitro infection models, Bd (2.5 × 10^5) and Bsal (7.5 × 10^5) zoospores were seeded on coverslips in infection medium 40% for Bd and 30% for Bsal with or without the addition of sublethal concentrations of 1,10-Phenanthroline (25–50 µM). As a control 0.005% v/v PI 8215 and PI 8340 were included. After 2 h, the medium was changed to 70% infection medium with or without the same PI treatment. To assess the recovery capacity, at 4 h post-treatment, zoospores were gently washed with water and 70% infection medium was added. After 16 to 24 h, the zoospores were also stained with CFW (50 µg/ml, 10 min). After CFW staining, the zoospores were fixed for 10 min with 3.7% paraformaldehyde in 70% HBSS + and mounted with ProLong™ Gold antifade mountant. Observations were qualitative only and no quantitative analysis was performed, as the assay served solely to illustrate the effects of the inhibitors on germ tube formation.

### Detection of protease activity

The influence of 1,10-Phenanthroline exposure on protease activity of Bd and Bsal zoospores was assessed according to [44]. In short, zoospores were harvested from 175 cm^2^ cell culture flasks by replacing the TGhL broth with distilled water, which was filtered using a sterile mesh filter with a pore size of 10 μm (Pluristrainer, PluriSelect). A pool containing approximately 10^7^ zoospores/ml was obtained. Two hundred microliters of the spore suspension was added to eppendorfs containing 200 µl H_2_O, 200 µl 2 mM 1,10-Phenanthroline, 200 µl 200 µM 1,10-Phenanthroline or 200 µl 40 µM 1,10-Phenanthroline. After 2 hours at 15 °C (Bsal) or 20 °C (Bd), the zoospores were centrifuged for 5 min at 4.000 × g, and the supernatant was collected. Protease activity in the supernatant was analyzed via a Pierce Fluorescent Protease Assay Kit (Thermo Fisher Scientific) according to the manufacturer’s instructions. Three independent repeats of the experiment were performed (biological replicates), with two technical replicates per experiment. In the figures, data points represent the means of the technical replicates for each biological replicate.

### Influence of divalent cations on the effect of 1,10-Phenanthroline on Bd and Bsal growth

To evaluate the impact of divalent cations on the effect of 1,10-Phenanthroline on chytrid viability, Bd (2.5 × 10^5^) and Bsal (7.5 × 10^5^) zoospores were seeded in TGhL medium containing varying concentrations of 1,10- Phenanthroline (100, 20 or 4 µM) and/or 20 mM CaCl_2_ or 20 mM MgCL_2_ in 24-well plates [15]. ZnCl_2_ was also tested, however, at 100 µM in combination with 1,10- Phenanthroline (20 or 100 µM), since higher ZnCl_2_ concentrations precipitated or negatively influenced chytrid growth (Supplementary Figure S6). As a control, zoospores were treated with TGhL or TGhL supplemented with DMSO at the highest concentration used in the treatments to account for any solvent effect. After 5 days of exposure, growth was assessed by taking pictures. The medium was then replaced with 1 ml of control TGhL medium, after which cultures were further incubated for 4 more days in the absence of the protease inhibitor, corresponding to 9 days p.i. Finally, Bd and Bsal growth was examined microscopically and imaged, a method more sensitive than qPCR for detecting partial or transient growth effects. Images were captured via a microscope equipped with a 20x objective lens and a 10x eyepiece, resulting in a total magnification of 200×. In addition to microscopy, images were analyzed using ImageJ software (1.54p) to quantify fungal growth. Thresholding was applied to each image to isolate fungal structures, and the percentage of the image area covered by Bd or Bsal (% area) was calculated as a quantitative proxy for growth. All treatments were tested in at least three independent experiments (biological replicates) with two technical replicates. In the figures, data points represent the means of the technical replicates for each biological replicate, with error bars indicating the SEM of the technical replicates.

### Assessment of the effects of 1,10-Phenanthroline on Bsal colonization via an *ex vivo* skin model

To evaluate the effect of 1,10-Phenanthroline on Bsal colonization of amphibian skin, an *ex vivo* infection model was established via skin biopsies from three adult *P*. *waltl*. The animals used in this study were captive-bred and maintained at the animal facility of the Faculty of Veterinary Medicine (Ghent University). Husbandry and euthanasia methods were in accordance with the guidelines of the Ethical Committee of the Faculty of Veterinary Medicine (Ghent University). Animals were euthanized by intracoelomic injection of sodium pentobarbital (Annex IV of the EU directive 2010/63), which ensures rapid loss of consciousness and death in a single step [38]. From each animal, 5 mm diameter, full-thickness ventral skin biopsies were aseptically collected. For each treatment condition, biopsies were processed in triplicate. Bsal zoospores were collected from mature cultures in sterile distilled water. The suspension was filtered through a 10 μm sterile filter to remove sporangia and adjusted to 10⁶ zoospores/mL. Skin biopsies were transferred to 24-well plates and exposed to 250 µL of Bsal zoospore suspension with or without 1,10-Phenanthroline (final concentration of 20 µM or 100 µM) in a total volume of 500 µL H_2_O. Plates were incubated at 15 °C. At 24 h p.i., a subset of samples was washed three times with sterile distilled water and harvested for DNA extraction and Bsal-specific qPCR. The remaining samples were gently washed twice with H_2_O and finally supplemented with 500 µl fresh H_2_O either at 24 h p.i. or at 3 days p.i. On day 5 p.i., all biopsies were washed and harvested for DNA extraction. DNA was extracted using the DNeasy Blood & Tissue Kit (Qiagen), followed by Bsal quantification using qPCR as described by Blooi et al. [4]. All the qPCRs were performed in duplicate using 1/10 diluted DNA. Genomic equivalents (GE) were determined based on a standard curve, and total loads were calculated by correcting for the 1/10 dilution and the total DNA volume.

### Statistical analysis

 First, to assess the effect of treatment (PI 8340, PI 8215 or 1,10-Phenanthroline) concentration on Bd and Bsal loads (GE per well), we fitted a generalized linear mixed model (GLMM) with a negative binomial distribution to account for overdispersion in the count data (glmmTMB package, [6]). We evaluated both nbinom1 and nbinom2 parameterizations and selected the final model based on lower Akaike Information Criterion (AIC) values. Models included concentration as a fixed effect and we included random intercepts for technical replicates nested within biological replicates. Second, to evaluate the effect of 1,10-Phenanthroline treatment on protease activity in Bd and Bsal, a linear mixed-effects model (LMM) was used with the lmer function (lme4 package, [2]). Protease concentrations (ng/mL) were log-transformed to meet model assumptions (normality of residuals and homoscedasticity). Models included concentration as a fixed effect and we included random intercepts for biological replicates. For both analyses, post hoc comparisons toward the negative control group were conducted via Dunnett’s test for multiple comparisons via the multcomp package [17]. Adjusted *p*-values were reported via the single-step method to control for the familywise error rate. Third, percentage area measurements were analyzed via GLMMs with a beta distribution to account for the bounded nature of the response variable (% area) (glmmTMB package). Separate models were specified for Bd and Bsal. For Bd, the model included cation, 1,10-Phenanthroline concentration and time point as fixed effects, with an interaction between 1,10-Phenanthroline and time. For Bsal, the model included all interactions between cations, 1,10-Phenanthroline and time points. In both models, random intercepts were included for technical replicates nested within biological replicates. For pairwise comparisons at each time point, estimated marginal means (EMMs) were calculated via the emmeans package [20]. Bonferroni correction was applied to adjust for multiple comparisons. Finally, the *ex vivo* infection data were analyzed via LMM applied to log-transformed Bsal loads (log₁₀(Total loads + 1). The model included 1,10-Phenanthroline concentration, time point (24 h p.i., 5 days p.i.: medium change 24 h p.i. or. 5 days p.i.: medium change 3 days p.i.), and their interaction as fixed effects, and random intercepts for individual animals. Post hoc comparisons of EMMs were performed via the emmeans package with Bonferroni correction. All statistical analyses were performed in R (version 4.2.3), and significance was assessed at α = 0.05. All models were checked for assumptions, residual distributions, random effect structures, and overall fits. The outputs of the models are summarized in Supplementary Table 1.

## Results

### 1,10-Phenanthroline suppresses spore development and germ tube formation required for infection progression

**Fig. 1:**
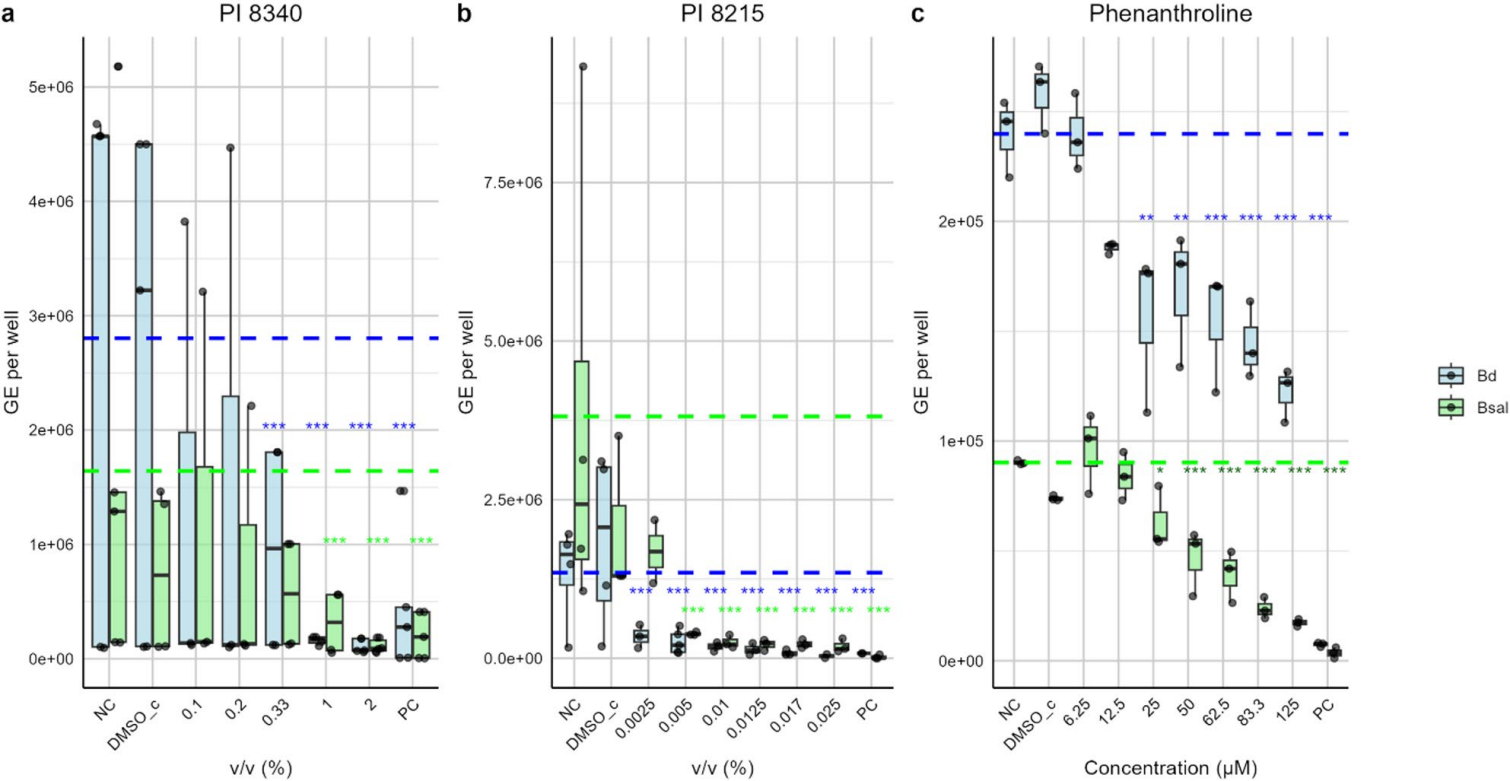
Effects of protease inhibitors on Bd and Bsal viability. The growth of Bd (blue) and Bsal (green) zoospores, expressed as genomic equivalents (GE) per well, was assessed after exposure to varying concentrations of (**a**) PI 8340, (**b**) PI 8215, and (**c**) 1,10-Phenanthroline for 24 h. Following exposure, the medium was replaced with TGhL, and growth was measured 5 days p.i. Horizontal dashed lines indicate the mean GE of the negative control (NC). NC = TGhL-treated zoospores; PC = heat-killed zoospores; DMSO_c = zoospores treated with TGhL supplemented with DMSO as a solvent control. Results are shown as box plots of biological replicates (jittered points), with boxes representing the 25th and 75th percentiles, central lines indicating the median and whiskers extending to 1.5× the interquartile range. Statistically significant differences compared with the NCs are indicated (*p ≤ 0.05; **p ≤ 0.01; ***p ≤ 0.001)

The impact of PIs on Bd and Bsal zoospore dynamics revealed distinct differences among the tested compounds (Fig. 1). PI 8340 exhibited minimal toxicity, with zoospore viability remaining high across most concentrations. Significant reductions in viability were observed only at concentrations ≥ 0.33% (v/v) for Bd and ≥ 1% (v/v) for Bsal (Fig. 1a), with an EC_50_ of 0.35% (v/v) in A6 cells (Supplementary Figure S1). Exposure to sub-EC_50_ concentrations of PI 8340 (0.2% v/v) during the first 24 h of infection did not appear to prevent Bd or Bsal invasion or maturation in A6 cells, with infected cells showing patterns similar to those of untreated controls, characterized by germ tube formation and subsequent intracellular penetration of A6 cells (Supplementary Figure S2). Actin staining further confirmed active host cell invasion by both Bd and Bsal, revealing germ tube penetration coinciding with localized actin assembly around the invading germ tube and intracellular transfer of chytrid contents (Supplementary Figure S3). These findings indicate that PI 8340 has little effect on Bd and Bsal growth and invasion under the conditions tested.

In contrast, PI 8215, which includes the metalloprotease inhibitor 1,10-Phenanthroline at a concentration of 500 mM, had pronounced effects on zoospore viability. A 24-hour treatment significantly impacted Bd and Bsal viability at concentrations as low as 0.0025% (v/v) (corresponding to 12.5 µM 1,10-Phenanthroline) and 0.005% (v/v) (corresponding to 25 µM 1,10-Phenanthroline), respectively, approximately 10-fold lower than its EC_50_ of 0.032% (v/v) in A6 cells (Fig. 1b, Supplementary Figure S1). Testing 1,10-Phenanthroline alone confirmed these effects, with dose-dependent reductions in viability observed for Bd and Bsal at concentrations ≥ 25 µM (Fig. 1c). These findings suggest that removal of divalent cations during the critical early period after attachment is either lethal to the zoospores or strongly delays their development.

**Fig. 2:**
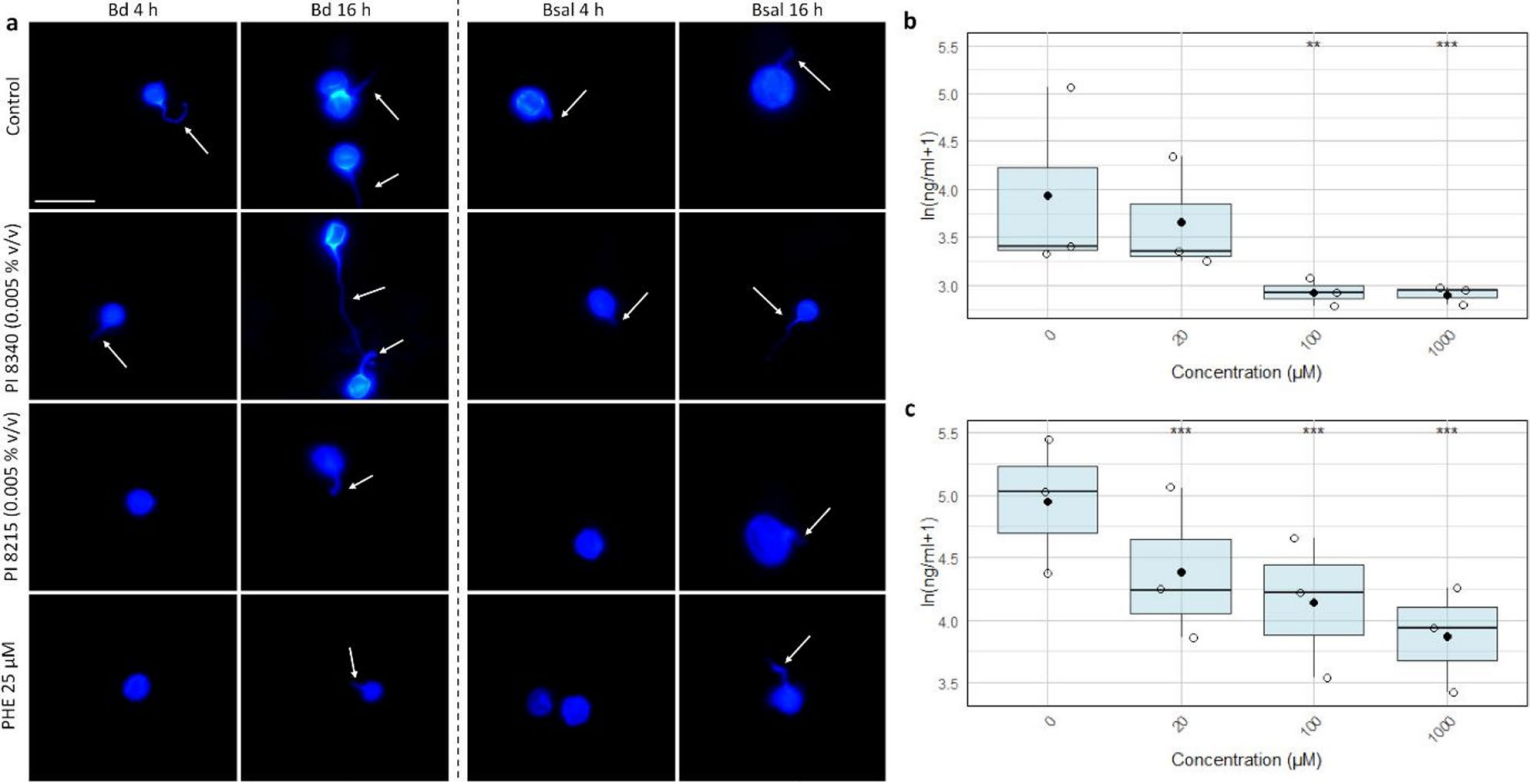
Inhibition of germ tube development and secreted protease activity in Bd and Bsal by PIs. (**a**) Representative CFW images of Bd and Bsal zoospores treated with PI 8215 (0.005% v/v), PI 8340 (0.005% v/v), and 1,10-Phenanthroline (25 µM) for 4 h, after which the medium was changed to unsuplemented TGhL for another 12 h. White arrows indicate germ tube formation. Scale bar = 10 μm. (b, c) Protease activity (ng/ml) was measured in the supernatants of (**b**) Bd and (**c**) Bsal zoospores incubated for 2 h with 1,10-Phenanthroline (0 (negative control, NC), 20, 100 and 1000 µM). Protease concentrations are shown as ln(ng/ml + 1). Data are shown as boxplots of biological replicates (jittered points), with boxes representing the 25th and 75th percentiles, central lines indicating the median, whiskers extending to 1.5× the interquartile range and black diamonds indicating the mean. Statistically significant differences compared with the NCs are indicated (*p ≤ 0.05; **p ≤ 0.01; ***p ≤ 0.001)

To investigate this more directly, protease activity was measured during the first 2 h after spore collection. Both Bd and Bsal zoospores secreted proteases as early as 2 h after collection, and this activity was strongly inhibited by 1,10-Phenanthroline in a dose-dependent manner (Fig. 2b-c). For Bd, significant inhibition occurred at 100 and 1000 µM, whereas Bsal activity was already significantly reduced at 20 µM. This reduction in Bsal protease activity was observed at a sublethal concentration, indicating that the effect is unlikely to be solely due to reduced zoospore viability, whereas at higher concentration and particularly for Bd, a contribution of reduced viability cannot be excluded. Together, these results demonstrate that chytrid zoospores actively secrete metalloproteases shortly after their release from sporangia and that this enzymatic activity can be efficiently inhibited by chelation of divalent cations, at least in the case of Bsal.

At sublethal concentrations (25–50 µM), a 24-hour exposure to 1,10-Phenanthroline significantly reduced fungal growth, as measured by qPCR (Fig. 1c), yet zoospores remained viable and could resume development once the inhibitors were removed (Supplementary Figure S4). Morphologically, germ tube formation was suppressed within 4 h of PI 8215 (0.005% v/v) or 1,10-Phenanthroline (25 µM) treatment, but recovery experiments revealed that elongation and further development resumed after 12 h in fresh medium following medium replacement (Fig. 2a, Supplementary Figure S5). These findings indicate that, at sublethal concentrations, metalloprotease inhibition suppresses spore development without causing irreversible damage. Under these conditions, progression is temporarily halted, but once divalent cations become available again, zoospores are able to resume germ tube formation and continue development. Consistent with these observations, treatment of Bd- or Bsal-infected A6 cells with PI 8215 (0.005% v/v) or 1,10-Phenanthroline (25 µM) similarly suppressed early germ tube formation and intracellular penetration at day 1 p.i., however, following medium replacement, signs of intracellular invasion were again observed at day 5 p.i., indicating recovery of infective progression in the host cell model (Supplementary Figure S2). Taken together, these data support an important role for metalloproteases in the earliest stages of Bd and Bsal development. Their activity is detectable within hours after spore collection, is required for germ tube formation, and thus represents a critical step in progression to infective stages.

### Cation availability modulates metalloprotease-dependent spore development

**Fig. 3:**
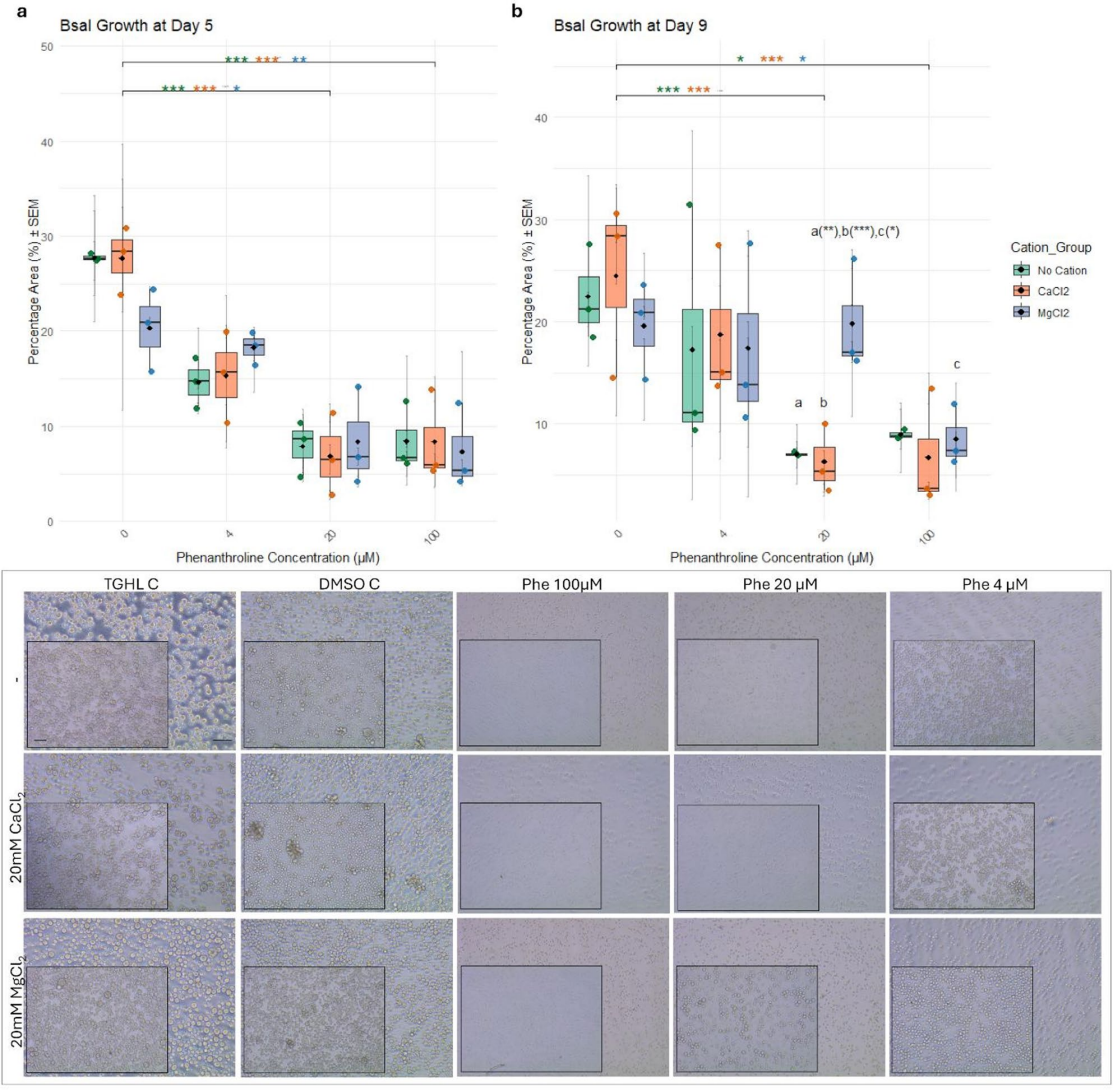
Recovery of Bsal zoospores following 1,10-Phenanthroline treatment in the presence of CaCl_2_ and MgCl_2_. (**a**) The mean percentage area ± SEM per biological replicate covered by fungal structures was calculated to assess Bsal zoospore growth after treatment with varying concentrations of 1,10-Phenanthroline (100 µM, 20 µM, 4 µM, or 0 µM), either alone or in combination with divalent cations (20 mM CaCl_2_ or 20 mM MgCl_2_), for 5 days p.i. (**b**) Quantification of Bsal growth on day 9 p.i., following replacement of the treatment medium with unsupplemented TGhL on day 5, to assess recovery and viability after 4 additional days in the absence of the protease inhibitor. Data are shown as boxplots of biological replicates (jittered points), with grey error bars representing the SEM of technical replicates, with boxes representing the 25th and 75th percentiles, central lines indicating the median, black whiskers extending to 1.5× the interquartile range and black diamonds indicating the mean. Statistically significant differences between 1,10-Phenanthroline treatment groups are indicated with *p ≤ 0.05; **p ≤ 0.01; ***p ≤ 0.001, with asterisk colors corresponding to specific comparisons. The same letters (a, b or c) denote relevant significant differences between particular 1,10-Phenanthroline conditions. (Lower panel) Representative microscopy images of Bsal zoospores under the indicated treatments on day 5 p.i. (background images) and day 9 p.i. (detailed images). Controls include TGhL medium alone (TGhL C) and TGhL supplemented with DMSO as a solvent control (DMSO C). The “-” indicates no addition of divalent cations. Scale bar = 50 μm

**Fig. 4:**
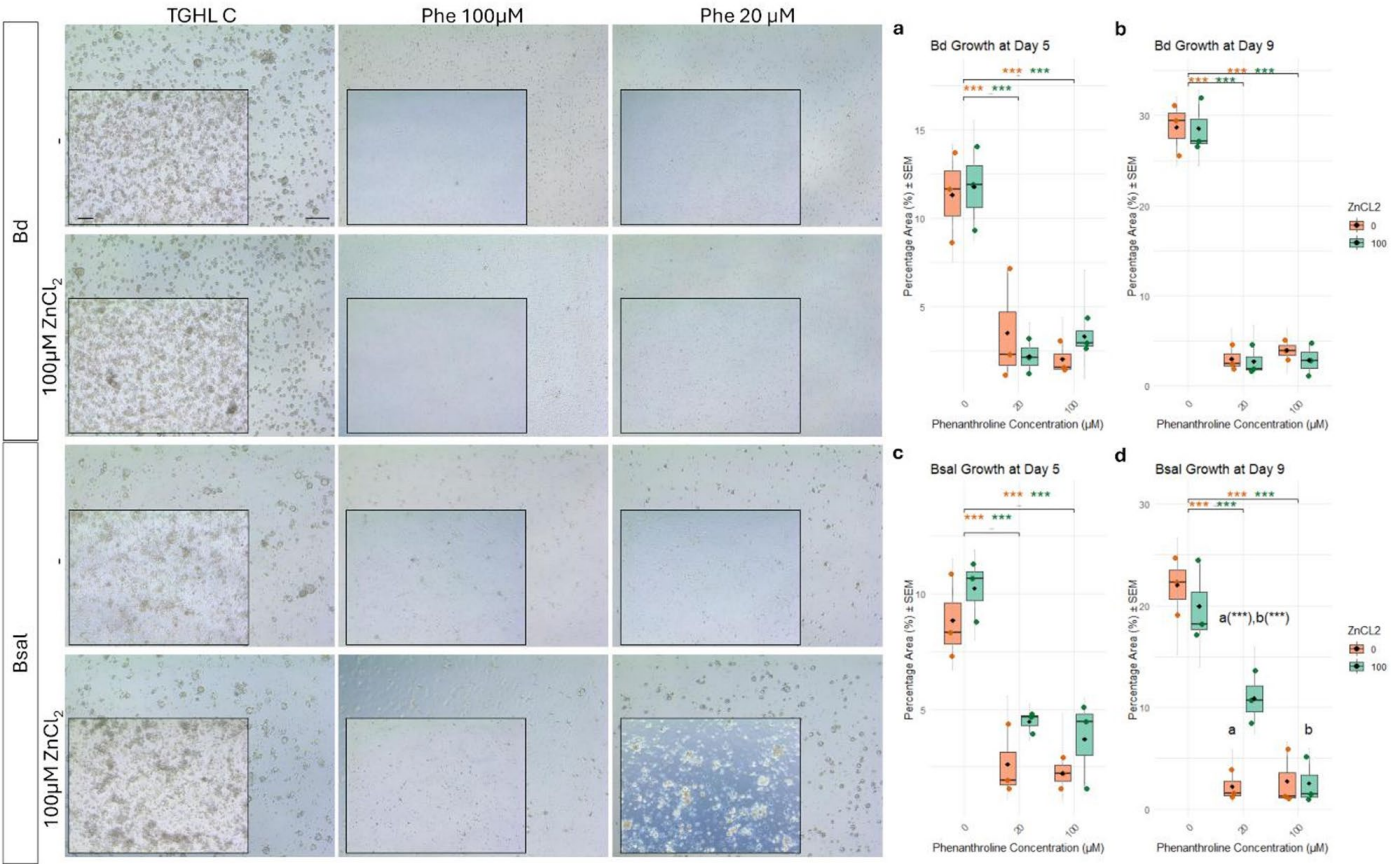
Recovery of Bd and Bsal zoospores following 1,10-Phenanthroline treatment in the presence of ZnCl_2_. The mean percentage area ± SEM per biological replicate covered by fungal structures was calculated to assess (**a**) Bd and (**c**) Bsal zoospore growth after treatment with varying concentrations of 1,10-Phenanthroline (100 µM, 20 µM, or 0 µM), either alone or in combination with 100 µM ZnCl₂ for 5 days p.i. Quantification of (**b**) Bd and (**d**) Bsal growth on day 9 p.i., following replacement of the treatment medium with unsupplemented TGhL on day 5, to assess recovery and viability after 4 additional days in the absence of the protease inhibitor. Data are shown as boxplots of biological replicates (jittered points), with grey error bars representing the SEM of technical replicates, with boxes representing the 25th and 75th percentiles, central lines indicating the median, black whiskers extending to 1.5× the interquartile range and black diamonds indicating the mean. Statistically significant differences between 1,10-Phenanthroline treatment groups are indicated with *p ≤ 0.05; **p ≤ 0.01; ***p ≤ 0.001, with asterisk colors corresponding to specific comparisons. The same letters (a, b) denote relevant significant differences between particular 1,10-Phenanthroline conditions. (Left panel) Representative microscopy images of Bd and Bsal zoospores under the indicated treatments on day 5 p.i. (background images) and day 9 p.i. (detail images). Controls include TGhL medium alone (TGhL C) and TGhL supplemented with DMSO as a solvent control (DMSO C). The “-” indicates no addition of divalent cations. Scale bar = 50 μm

**Fig. 5:**
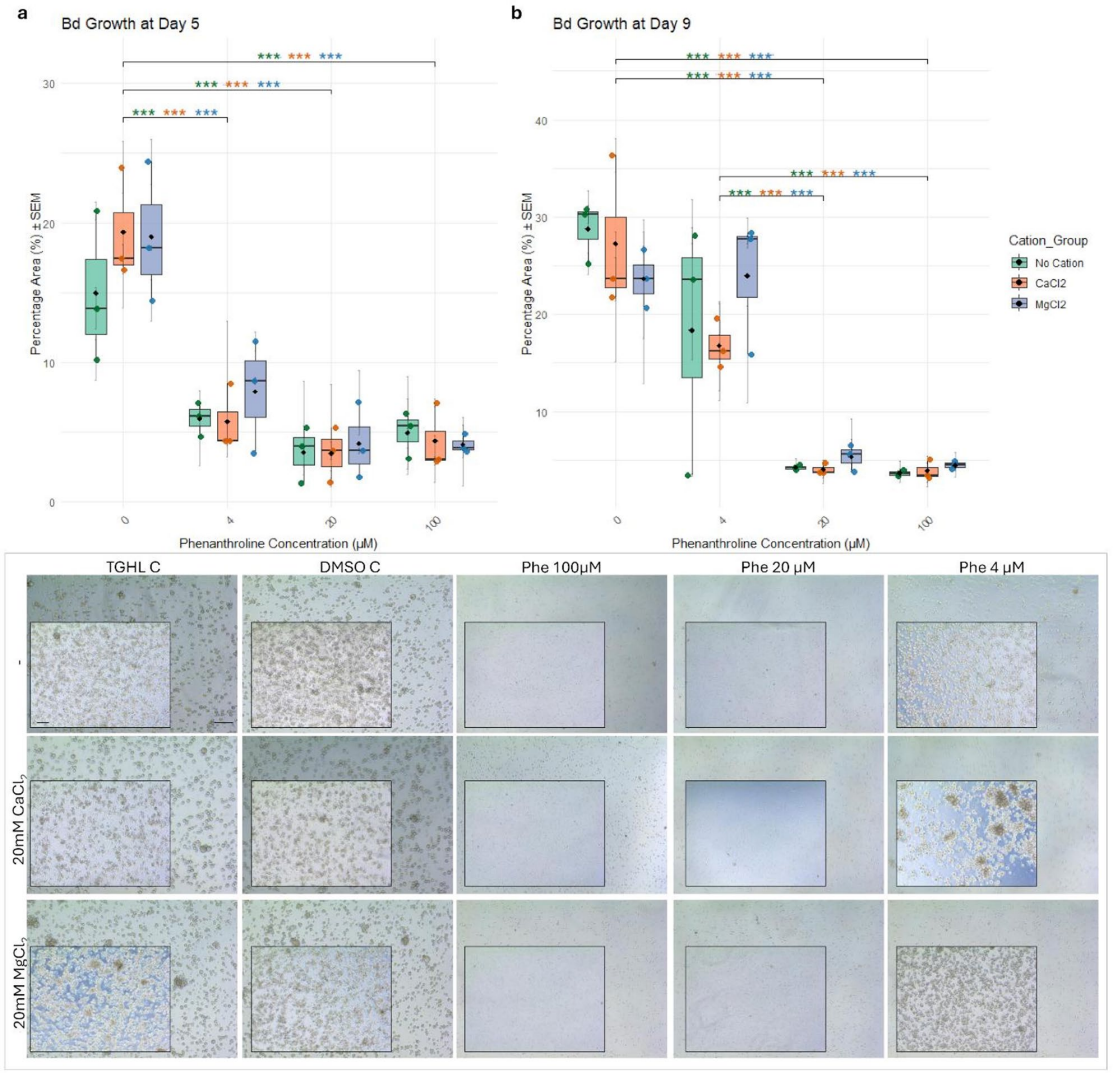
Recovery of Bd zoospores following 1,10-Phenanthroline treatment in the presence of CaCl_2_ and MgCl_2_. (**a**) The mean percentage area ± SEM per biological replicate covered by fungal structures was calculated to assess Bd zoospore growth after treatment with varying concentrations of 1,10-Phenanthroline (100 µM, 20 µM, 4 µM, or 0 µM), either alone or in combination with divalent cations (20 mM CaCl_2_ or 20 mM MgCl_2_), for 5 days. (**b**) Quantification of Bd growth on day 9 p.i., following replacement of the treatment medium with unsupplemented TGhL on day 5, to assess recovery and viability after 4 additional days in the absence of the protease inhibitor. Data are shown as boxplots of biological replicates (jittered points), with grey error bars representing the SEM of technical replicates, with boxes representing the 25th and 75th percentiles, central lines indicating the median, black whiskers extending to 1.5× the interquartile range and black diamonds indicating the mean. Significant differences between 1,10-Phenanthroline treatment groups are indicated with *p ≤ 0.05; **p ≤ 0.01; ***p ≤ 0.001, with asterisk colors corresponding to specific comparisons. (Lower panel) Representative microscopy images of Bd zoospores under the indicated treatments on day 5 p.i. (background images) and day 9 p.i. (detailed images). Controls include TGhL medium alone (TGhL C) and TGhL supplemented with DMSO as a solvent control (DMSO C). The “-” indicates no addition of divalent cations. Scale bar = 50 μm

1,10-Phenanthroline, a chelating agent that strongly binds metal ions, likely inhibits spore activity by sequestering these essential metal ions. To explore whether this inhibition could be counteracted, divalent cations were supplemented during prolonged exposure. A 5-day treatment with 20 µM or 100 µM 1,10-Phenanthroline resulted in significant Bsal growth inhibition, with no protection by Mg²⁺ or Ca²⁺ supplementation (Fig. 3a), and spores appeared small and morphologically undeveloped (Fig. 3, lower panel). Treatment with 4 µM 1,10-Phenanthroline for 5 days resulted in non-significant growth inhibition of Bsal (Fig. 3a). With Mg²⁺ supplementation, zoospores appeared more spherical and slightly rounded, indicative of partial resistance or altered development, although these differences were not statistically significant (Fig. 3a and lower panel). After removal of 1,10-Phenanthroline and incubation in normal TghL medium for 4 days (9 days p.i.), no recovery was observed in the 100 µM 1,10-Phenanthroline conditions (Fig. 3b). In contrast, significant growth recovery of Bsal was observed in the 20 µM 1,10-Phenanthroline condition if Mg²⁺ had been present during the initial 5-day treatment (Fig. 3b). Morphologically, this recovery was accompanied by the development of sporangia, indicative of an actively developing Bsal culture (Fig. 3, lower panel). At lower 1,10-Phenanthroline concentrations (4 µM), growth inhibition was reversible and occurred independently of cation supplementation (Fig. 3b and lower panel). A second experiment using Zn²⁺ strongly corroborated these findings. Exposure to 100 and 20 µM 1,10-Phenanthroline for 5 days markedly inhibited Bsal growth, and supplementation with Zn²⁺ did not alleviate this effect (Fig. 4c, left panel). Upon removal of 1,10-Phenanthroline and incubation in normal TghL medium for 4 additional days (9 days p.i.), cultures under the 100 µM condition failed to recover (Fig. 4d, left panel). By contrast, Bsal treated with 20 µM 1,10-Phenanthroline in the presence of Zn²⁺ during the initial 5 days exhibited restored growth at 9 days p.i. (Fig. 4d, left panel). These results confirm that the inhibitory effect of 1,10-Phenanthroline is mediated by depletion of essential divalent cations. Bd showed a somewhat different response to 1,10-Phenanthroline than Bsal. Five-day exposure inhibited growth at all tested concentrations (4, 20 and 100 µM), and co-treatment with divalent cations did not restore growth at higher concentrations (Figs. 4 and 5). At 4 µM, inhibition was reversible upon transfer to fresh medium, indicating transient suppression (Figs. 4 and 5).

**Fig. 6:**
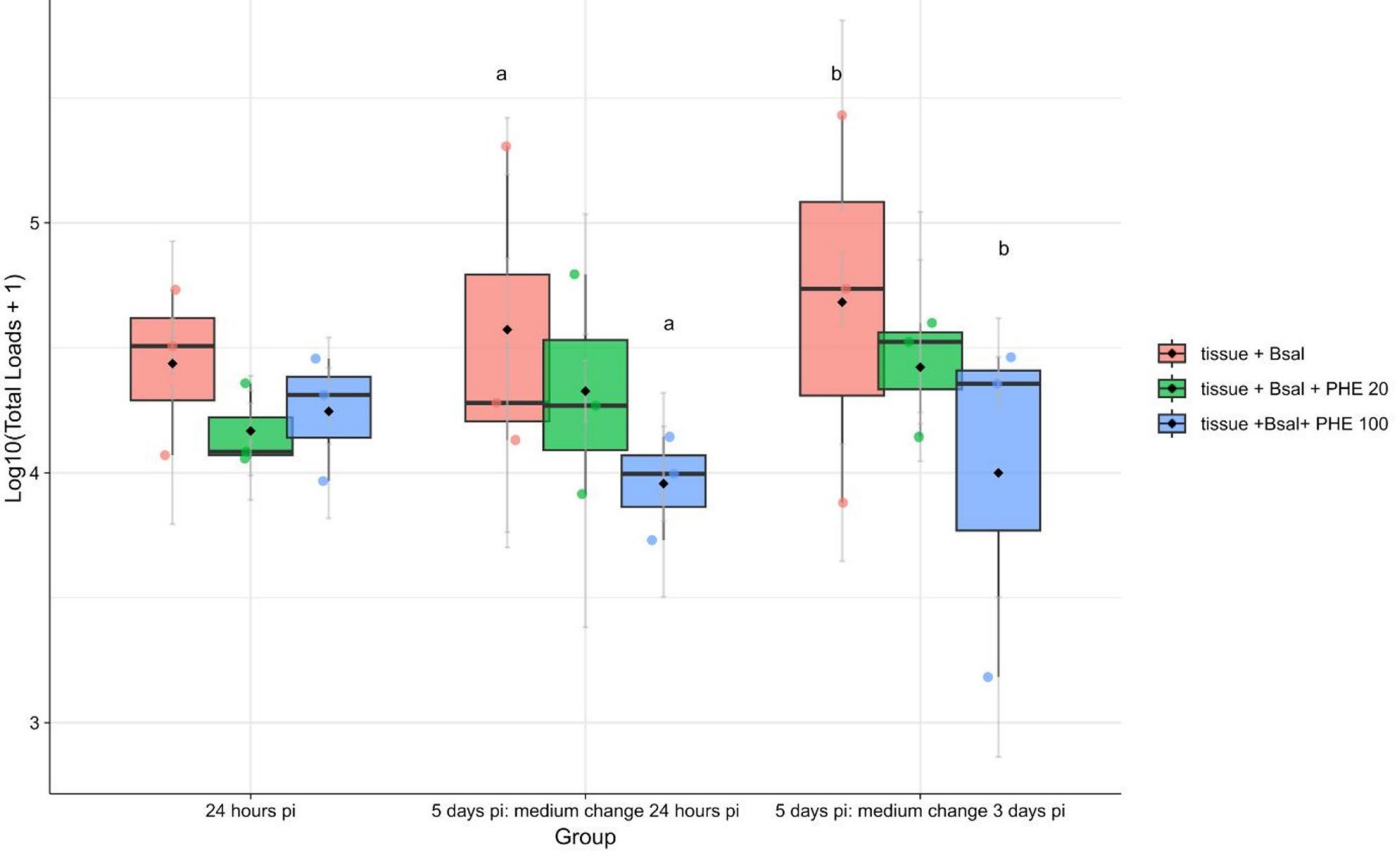
Bsal colonization of *Pleurodeles waltl* skin *ex vivo* with 1,10-Phenanthroline. Skin biopsies from three adult *P. waltl* were exposed in triplicate to Bsal zoospores in the absence (tissue + Bsal) or presence of 1,10-Phenanthroline at 20 µM (tissue + Bsal + PHE 20) or 100 µM (tissue + Bsal + PHE 100). Biopsies were harvested at 24 h post-inoculation (p.i.) or 5 days p.i. (with medium changed at either 24 h or 3 days p.i.), and Bsal loads were quantified via qPCR and log-transformed (log₁₀[total loads + 1]). Data are presented as boxplots of biological replicates (jittered points), with grey error bars representing the SEM of technical replicates (*n* = 3). Boxes represent the interquartile range (25th-75th percentile), central lines indicate the median, black whiskers extend to 1.5× the interquartile range, and black diamonds denote group means. Groups above the boxplots labeled with the same letter are considered different from each other: “a” corresponds to *p* = 0.0525, and “b” corresponds to *p* = 0.0261

To evaluate whether the observed effects translate to host tissue, we analyzed *ex vivo* Bsal infections of *P. waltl* skin. Treatment with 100 µM 1,10-Phenanthroline significantly reduced fungal load after both short (24 h) and prolonged (3 days) exposure (Fig. 6). The rapid effect observed within 24 h likely reflects disruption of essential metal-dependent processes during the earliest stages of spore development, including metalloproteases, with higher concentrations leading to lethal effects. In contrast, a sublethal concentration (20 µM) that impaired Bsal growth in vitro did not reduce fungal load *ex vivo* (Fig. 6). Based on our in vitro findings, such a concentration would be expected to transiently inhibit spore development and germ tube formation, with recovery occurring only after inhibitor removal and in the presence of divalent cations. The absence of any difference between treated and untreated samples therefore suggests that host-derived divalent cations immediately buffer the inhibitory effect, allowing zoospores to resume development, adhere to the epidermis, and invade host tissue. Taken together, these findings indicate that metal-dependent processes, including metalloprotease activity, play a central role in chytrid spore development and are tightly linked to divalent cation availability.

## Discussion

The differential sensitivity of Bd and Bsal to PIs underscores the critical role of specific protease classes in their pathogenicity. PI 8340, which targets serine, cysteine, and aspartyl proteases, showed minimal toxicity and, based on qualitative assessments, did not appear to alter zoospore invasion or maturation. Although TGhL broth is rich in peptides and amino acids, which could theoretically compete with or mask the activity of certain competitive inhibitors, such as Bestatin, the PI cocktails (PI 8340 and PI 8215) contain inhibitors at sufficiently high concentrations to maintain efficacy. Components that act irreversibly or non-competitively, such as AEBSF and E-64, are largely unaffected by substrate competition, suggesting that the minimal effects observed for PI 8340 reflect the limited role of these protease classes under the tested conditions rather than diminished inhibitor potency [11, 24, 35]. In contrast, PI 8215, which contains 1,10-Phenanthroline, strongly suppressed spore growth and germ tube formation at sublethal concentrations, and metalloprotease activity was detectable within two hours after spore collection. This demonstrates that metalloprotease activity is among the first processes activated during spore development and is functionally required for the transition from attachment to germ tube outgrowth. These early secretory events are in line with observations in other fungi, such as *Phialophora verrucosa*, where metalloprotease activity was detected during early developmental stages [15]. Our findings also provide an explanation for discrepancies with earlier work on chytrids. Farrer et al. [14] reported minimal protease activity in Bsal zoospores, but their measurements relied on lysed zoospores, which may underestimate the activity of secreted enzymes. As most M36 metalloproteases are exported [42], focusing on secreted fractions is more likely to capture their functional relevance.

The inhibition of spore development by 1,10-Phenanthroline hints towards the involvement of metalloproteases. At low 1,10-Phenanthroline concentrations (4 µM), zoospores of both Bsal and Bd can recover even in the absence of cations, suggesting that the effects on spore development are partial and reversible at this dose. In Bsal, supplementation with Mg²⁺ and Zn²⁺ partially restored spore activity in 20 µM 1,10-Phenanthroline-treated spores, indicating that zoospores are not irreversibly killed under inhibition. They remain viable but developmentally arrested when 1,10-Phenanthroline is present and can only resume growth and progress to sporangia after inhibitor removal. In contrast, growth recovery in Bd was not observed in 20 µM 1,10-Phenanthroline-treated spores after cation supplementation. The partial recovery of spore activity in Bsal upon cation supplementation is consistent with inhibition of metalloprotease-dependent processes. However, we cannot fully exclude that some of the observed suppression results from other phenanthroline-sensitive targets or broader metal-dependent processes. In addition to inhibiting metalloproteases, 1,10-Phenanthroline has been reported to affect other cellular processes, some related to metal chelation and others independent of it, including mitochondrial function, cell membrane integrity, and nuclear stability [25]. Future studies could employ alternative chelators with differing metal specificities, such as EDTA, or direct metalloprotease inhibitors like phosphoramidon, which binds Zn²⁺ in the active site. While phosphoramidon is primarily characterized for bacterial Zn²⁺-dependent metalloproteases, such as thermolysin, Monod et al. [27] and Farrer et al. [14] have successfully applied it to fungal metalloproteases. Incorporating such alternative chelators and direct inhibitors in future experiments could enable broader comparisons, allowing more precise distinction between metalloprotease-specific effects and general consequences of metal ion chelation. Overall, our findings underscore that modulation of metalloprotease activity likely contributes to controlling spore development and early infection stages, while acknowledging that additional metal-dependent processes may also influence these outcomes. This provides a mechanistic rationale for exploring metalloproteases as potential targets in pathogen control. Indeed, metalloprotease inhibitors have been investigated as therapeutic agents in other systems, including *Schistosoma mansoni* infections [10] and human diseases such as cancer [16]. These examples highlight the potential of metalloprotease-targeting strategies, but the associated risks of host toxicity and lack of specificity [43] emphasize the need for tailored inhibitors, with practical applications in chytrid infection likely confined to captive populations.

Zn²⁺ (100 µM) exerts a protective effect on Bsal at lower concentrations than Mg²⁺ (20 mM), possibly reflecting the fungus’s metalloprotease preferences. Metal ions, including Zn²⁺, are essential for both host and pathogen, supporting immune function in the host and virulence in the pathogen [1]. The fact that *ex vivo* infections are not suppressed at sublethal 1,10-Phenanthroline concentrations (20 µM) suggests that host-derived divalent cations buffer the chelating effects of 1,10-Phenanthroline [9]. This further supports the idea that metalloprotease activity likely contributes to spore development and germ tube formation in vitro and may also operate in the host environment, where metal availability could modulate the outcome. The common dependence of both the host and pathogen emphasizes Zn²⁺ as a key factor influencing infection outcomes. In addition to the essential requirement of Zn²⁺ for microorganisms, Zn²⁺ intoxication is also well recognized, whereby hosts exploit metal essentiality through nutritional immunity by either sequestering or overloading metals to restrict pathogen growth [1]. These findings are consistent with the growing body of evidence that environmental metal concentrations can influence chytrid infection dynamics. For example, amphibian populations have been reported to persist at metal-polluted sites where Bd is inhibited by Cu²⁺ and Zn²⁺ at concentrations tolerated by amphibians [12, 28]. This suggests that the interplay between metal availability, pathogen sensitivity, and host tolerance could shape infection outcomes in natural settings, with Zn²⁺ emerging as a central factor in both pathogen metabolism and host immunity.

## Conclusion

Our study suggests an important role for metalloproteases in the early stages of Bd and Bsal spore development and infectivity. The partial rescue by divalent cations in Bsal underscores how metal availability can directly influence pathogen virulence, revealing that environmental and host-associated metal dynamics may shape infection outcomes in amphibians. These findings provide mechanistic insight into chytrid pathogenicity and the factors governing early host colonization.

## Supplementary Information

Supplementary Material 1.

Supplementary Material 2.

## Data Availability

All data generated or analyzed during this study are included in this published article and its supplementary information files.
