# Supplementary material for: Infectivity of amphibian chytrid fungi requires metalloprotease-driven spore development and germ tube formation
Source: BMC Microbiol. 2026 Mar 25;26:428. doi: 10.1186/s12866-026-04896-x (PMC13137486; doi:10.1186/s12866-026-04896-x)
Supplement: Supplementary file 2 — Supplementary Material 2. [file 12866_2026_4896_MOESM2_ESM.docx]

**Supplementary Figures:**


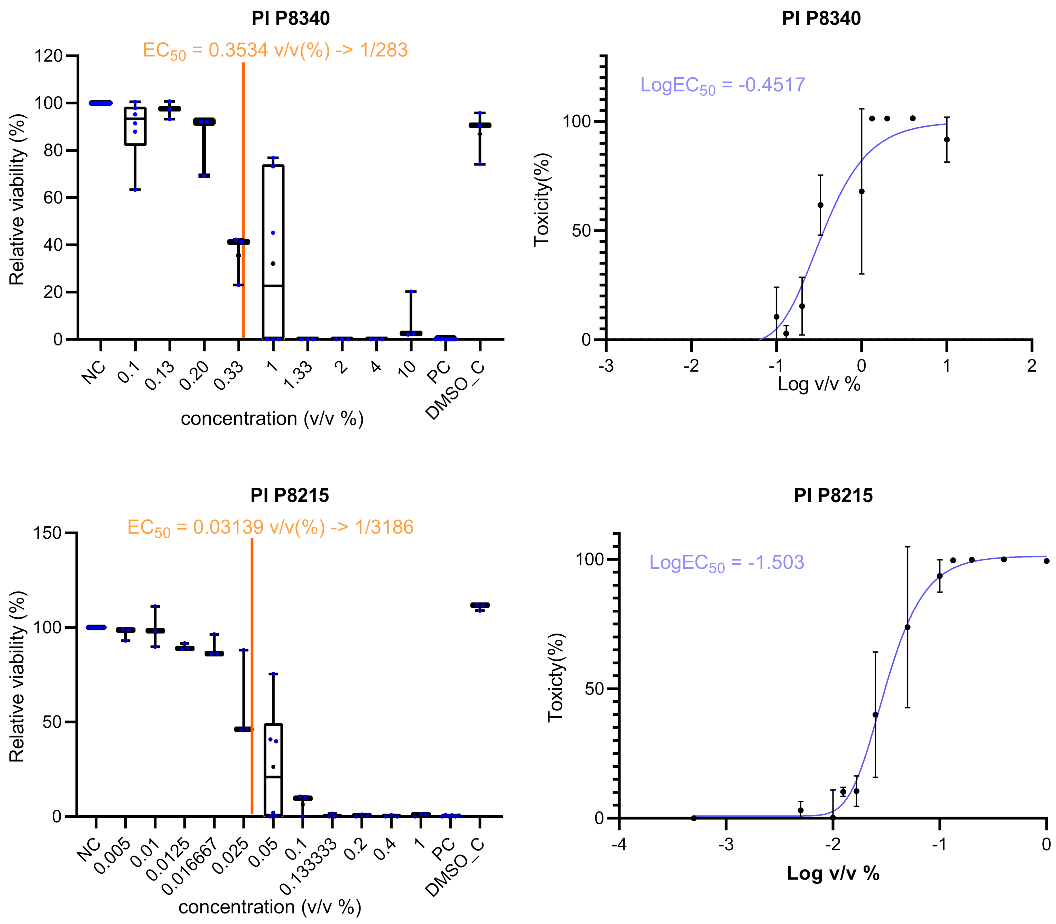


**Figure S1:** Effect of PI’s on the viability of A6 cells. **(Left Panels)** Relative viability (%) of A6 cells treated with PI P8340 or PI P8215 for 24 hours, determined by neutral red assay. NC = untreated A6 cells or negative control; PC = A6 cells treated with 1% triton (Triton_C); DMSO = solvent control (DMSO_C). EC_50_ is depicted by the orange line. Results are shown as box plots with boxes indicating the 25^th^ and 75^th^ percentiles, central lines the median, bars the minima and maxima and points indicate the biological replicates**.** (**Right Panels**) Dose dependent sigmoidal toxicity curve that was used to determine the EC_50_.


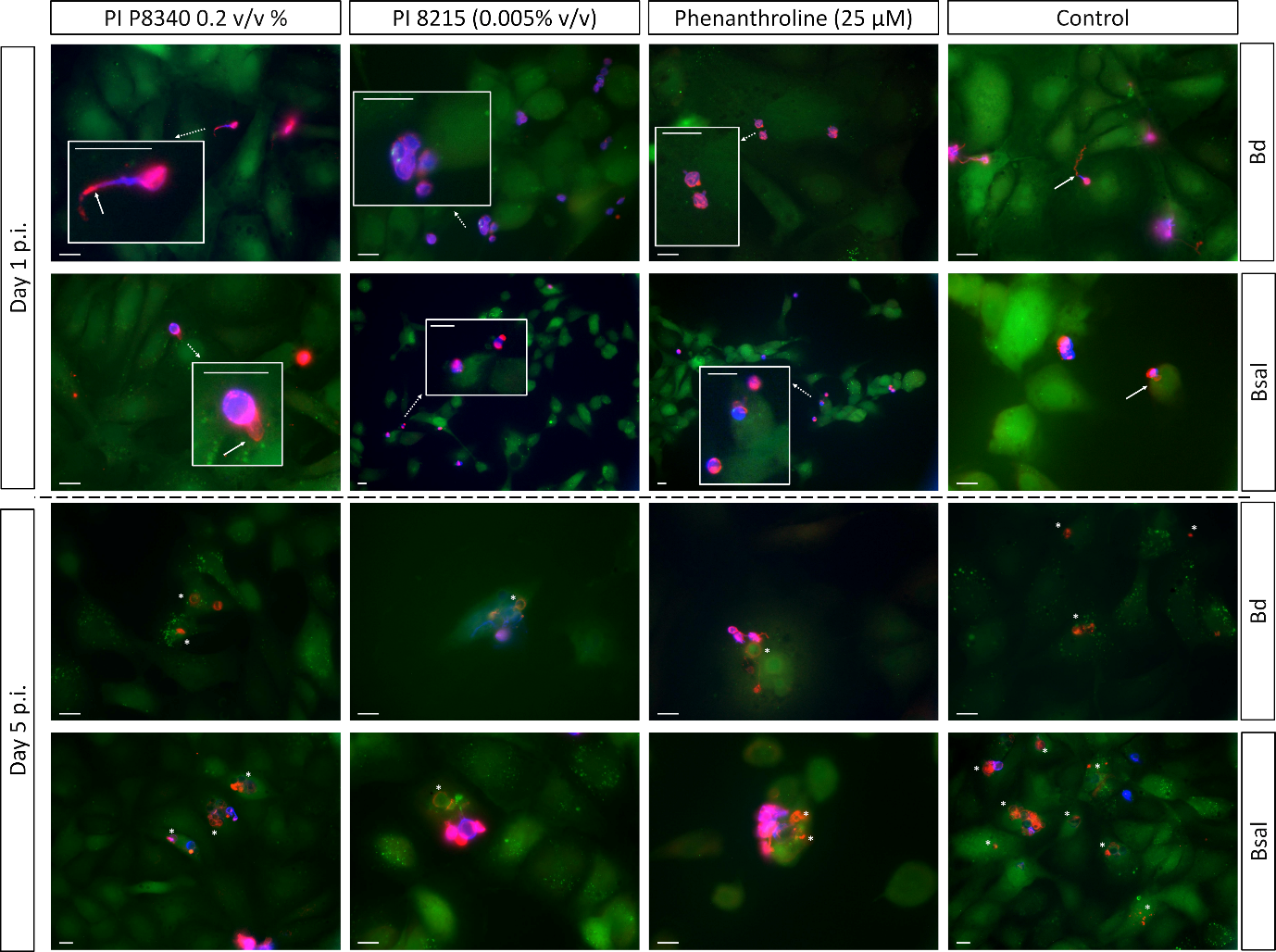


**Figure S2:** Chytrid-A6 cell following transient protease inhibitor treatment. Fluorescent overlay images depict Bd- or Bsal-infected A6 cells at day 1 and 5 p.i. with or without addition of PI P8340 (0.2 v/v %), PI P8215 (0.005 v/v%) and 1,10-Phenanthroline (25 µM) during the first 24 hours of interaction. After 24 hours, the medium was replaced with inhibitor-free control medium, and cultures were further incubated until day 5 p.i. Representative figures are shown of chytrid-infected A6 cells (CellTracker Green), extracellular chytrid (Calcofluor White, blue), and extra- and intracellular chytrid (Alexa Fluor 568, red). Details are indicated by dashed arrows and shown in enlarged panels. At day 1 p.i., germ tube formation with intracellular penetration (solid white arrows) is observed in control and PI P8340-treated conditions, but not in PI P8215- or 1,10-Phenanthroline-treated samples. At day 5 p.i. (four days after inhibitor removal), signs of Bsal internalization are observed in all conditions (white asterisks), indicating recovery of Bsal spores following transient exposure to sublethal concentrations of PI P8215 and 1,10-phenanthroline. Scale bar = 10 µm.


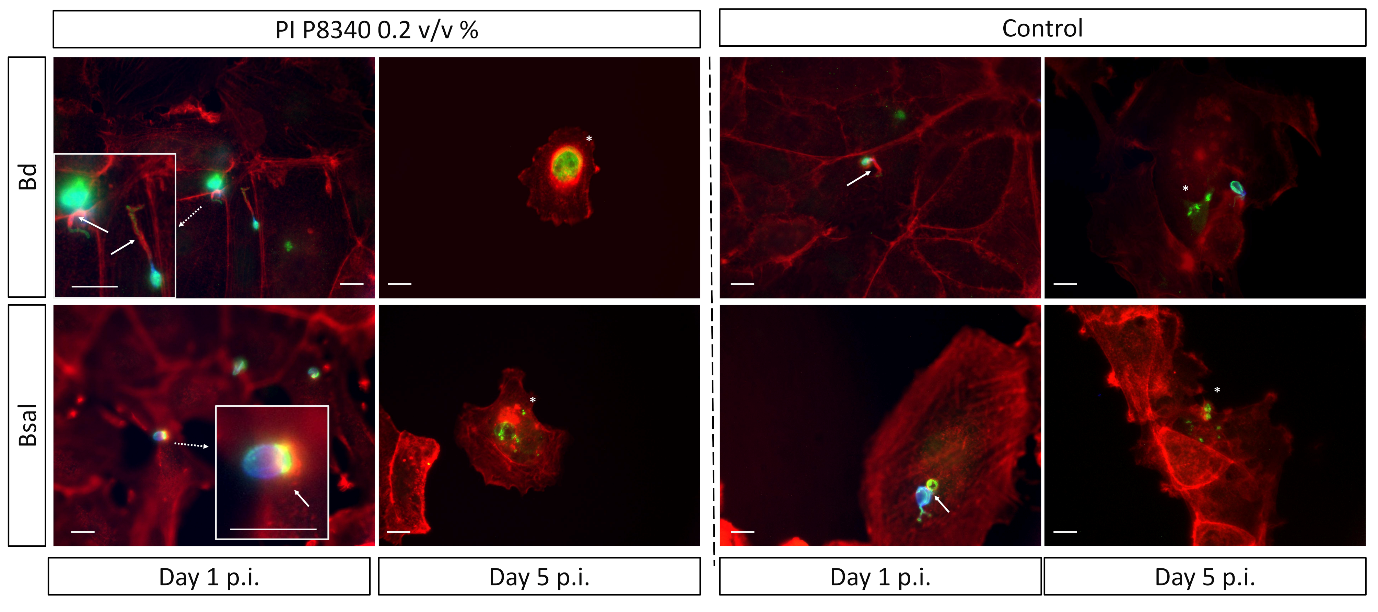


**Figure S3. Actin-associated chytrid invasion of A6 cells following transient PI P8340 treatment.**Fluorescent overlay images show Bd-or Bsal-infected A6 cells at day 1 and day 5 p.i. During the first 24 hours of host-pathogen interaction, cultures were treated with PI P8340 (0.2 v/v%) or left untreated. After 24 hours, the medium was replaced with inhibitor-free control medium, and cultures were further incubated until day 5 p.i.

Images depict the A6 cell cytoskeleton stained with Phalloidin Texas Red (red), extra- and intracellular Bsal labeled with Alexa Fluor 488 (green), and extracellular Bsal stained with Calcofluor White (blue). Details are indicated by dashed arrows and shown in enlarged panels. Within 24 hours post-inoculation, both Bd and Bsal penetrate host cells, coinciding with actin assembly around the invading germ tube and intracellular transfer of chytrid contents (solid white arrows). At day 5 p.i., intracellular maturation of thalli is observed, accompanied by release of intracellular contents and loss of actin organization (white asterisks). Scale bar = 10 µm.


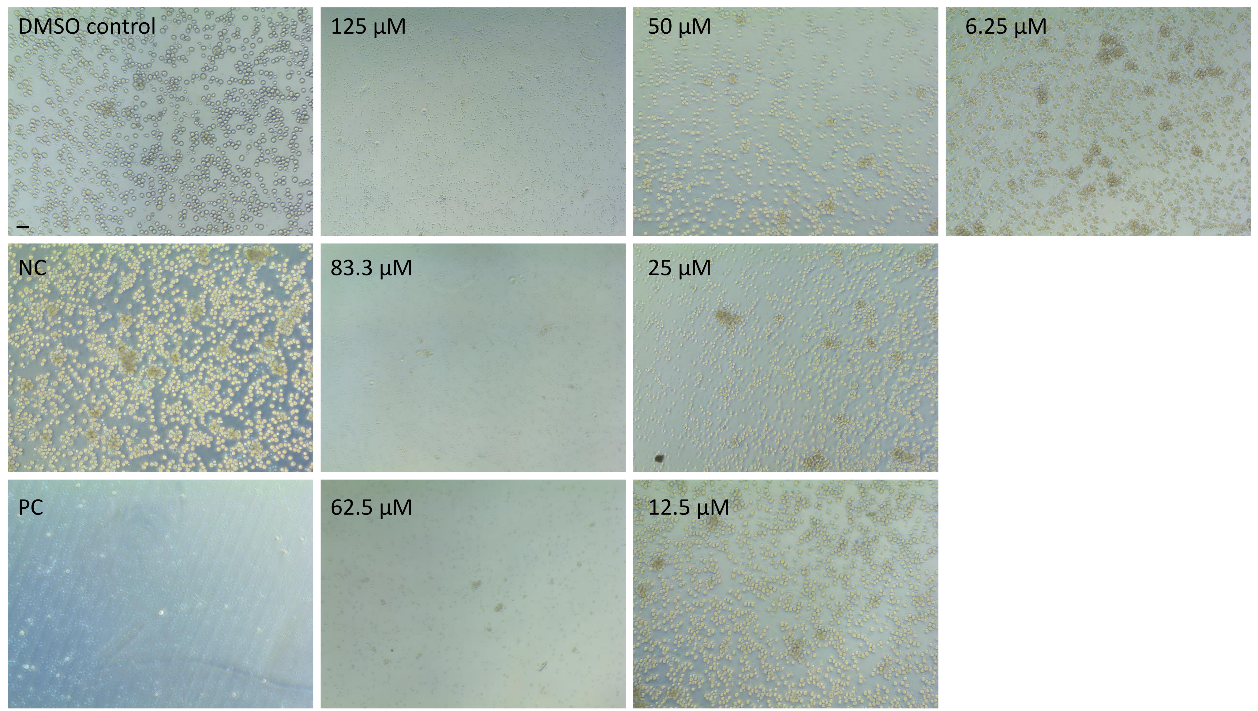


**Figure S4:** Effect of 1,10-Phenanthroline on Bsal growth. Representative images of Bsal zoospores treated with 1,10-Phenanthroline at various concentrations for 24 hours, followed by a medium change to TGhL. Growth was observed and imaged 5 days p.i. NC = TGhL-treated zoospores; PC = heat-killed zoospores; DMSO control = zoospores treated with TGhL supplemented with DMSO as a solvent control. Scale bar = 50 µm.


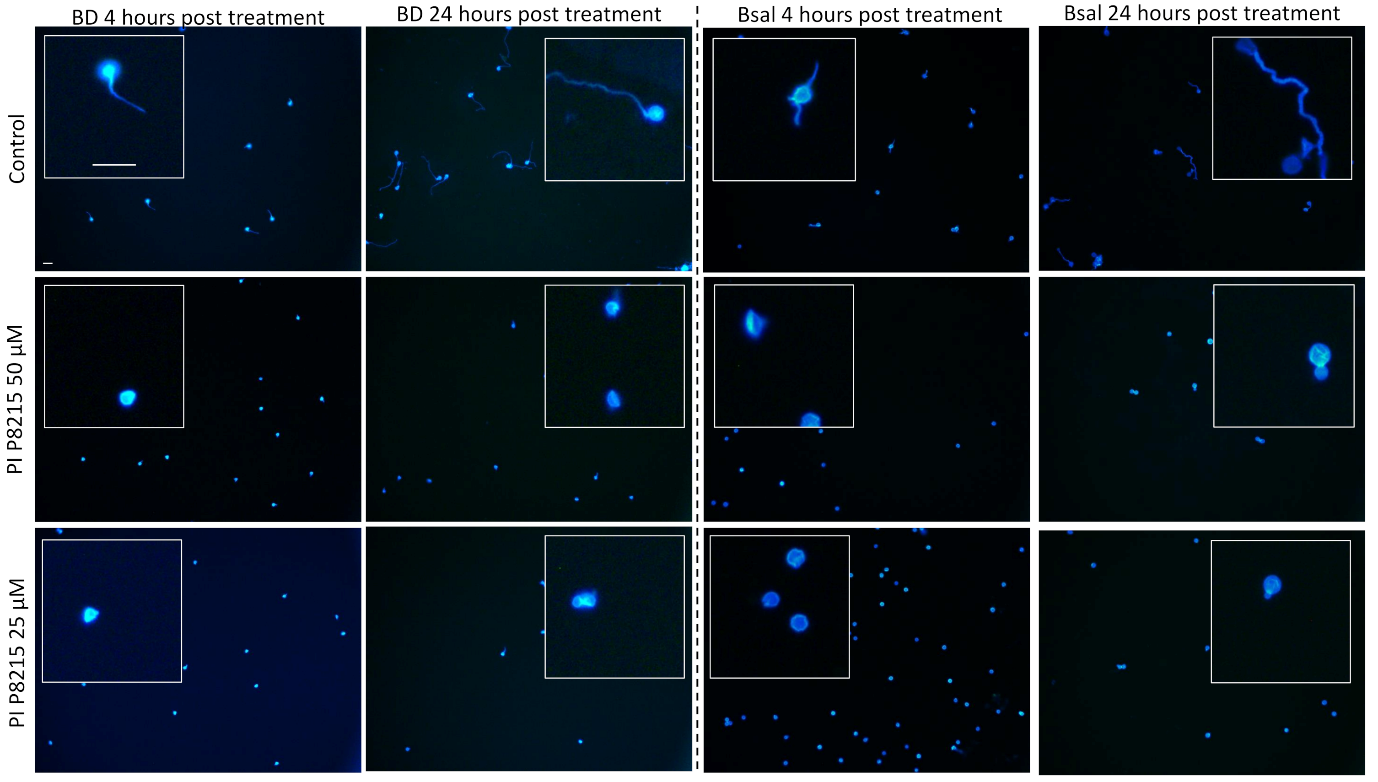


**Figure S5:** Influence of PI P8215 on Bd and Bsal germ tube development. Representative CFW images of Bd and Bsal zoospores treated with PI 8215 (50 and 25 µM) for 4 and 24 hours. Details highlighting the germ tube development, are depicted in the smaller detail pictures. Scale bar = 10 µm.

**
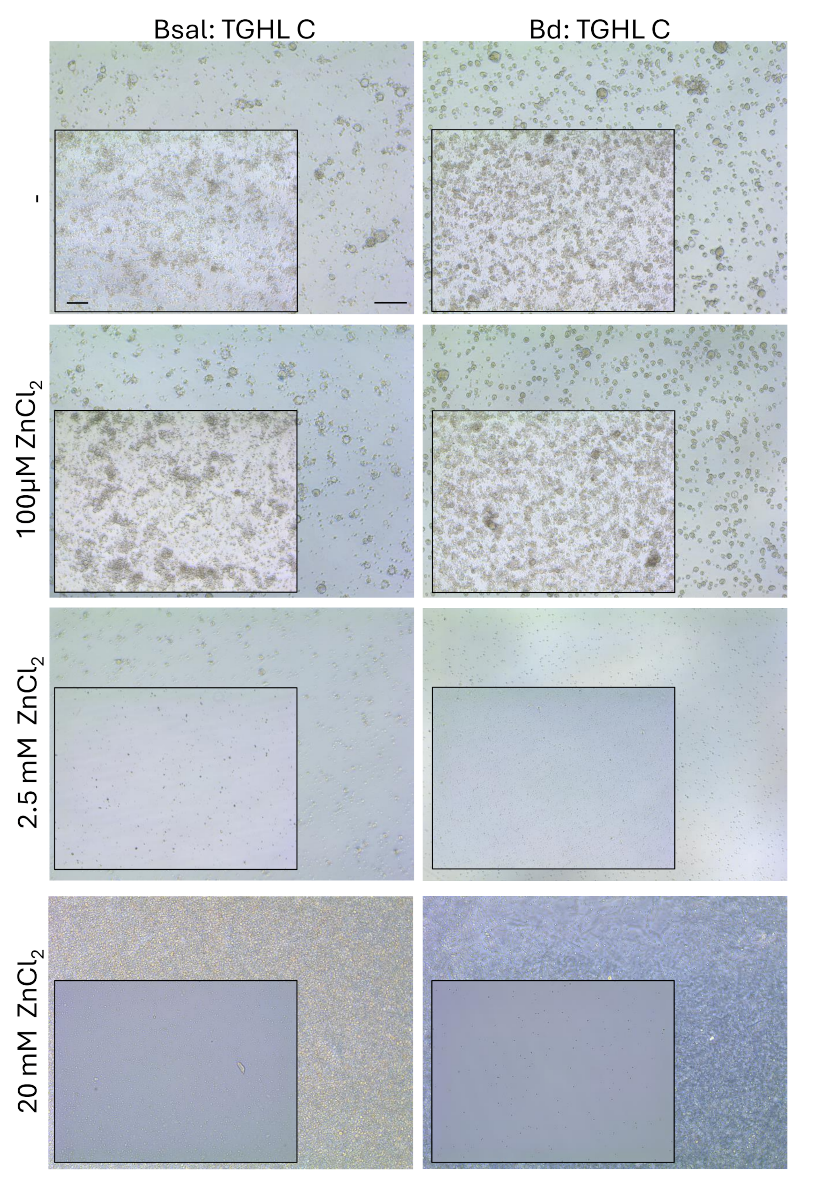
**

**Figure S6:** Precipitation and growth inhibition effects of ZnCl₂ in TGhL medium. Representative images showing the effect of ZnCl₂ concentrations on Bd and Bsal growth and precipitation in TGhL medium. At concentrations ≥ 2.5 mM, ZnCl₂ precipitated in the medium, which corresponded to growth inhibition. Concentrations ≤ 2.5 mM no longer caused precipitation but still inhibited growth. At 100 µM, ZnCl₂ did not precipitate or interfere with growth in control TGhL cultures. The "-" condition indicates the absence of ZnCl₂ supplementation. Scale bar = 50 µm.
